# Parallel mediating effects of anxiety and depression on the relationship between sleep quality and fear of progression in individuals recovering from COVID-19

**DOI:** 10.3389/fpsyg.2025.1528189

**Published:** 2025-04-30

**Authors:** Fang Chen, Ruiying Jia, Qiutang Wang, Min Li, Su Hong, Meijuan Lan, Leilei Zheng

**Affiliations:** ^1^Nursing Department, The Second Affiliated Hospital of Zhejiang University School of Medicine, Hangzhou, China; ^2^Department of Psychiatry, The Second Affiliated Hospital of Zhejiang University School of Medicine, Hangzhou, China

**Keywords:** sleep quality, anxiety, depression, fear of progression, post-COVID-19

## Abstract

**Background:**

The COVID-19 pandemic caused by the SARS-CoV-2 virus is one of the most significant public health emergencies of this century. The rapid outbreak of COVID-19 infections has instilled fear in populations. Therefore, it is essential to investigate the risk factors and mechanisms associated with fear of progression (FoP) among individuals recovering from COVID-19. This information is crucial for alleviating the physical and psychological discomfort of individuals after recovery and enhancing their long-term quality of life.

**Methods:**

A cross-sectional study involving 861 individuals recovering from COVID-19 was conducted in China from January to February 2023. The Pittsburgh Sleep Quality Index, the Self-Rating Depression Scale, the Self-Rating Anxiety Scale, and the Fear of Progression Scale were utilized to assess mood status, sleep quality, and fear of progression. Receiver operating characteristic curves, Pearson’s correlation analysis, binary logistic regression analysis, and Hayes’ PROCESS Macro analysis were employed to test the model.

**Results:**

The results showed that sleep quality, anxiety, depression, and fear of progression were positively correlated, with coefficients ranging from 0.380 to 0.814. After addressing potential bias, sleep quality, anxiety, and depression emerged as risk factors for fear of progression (FoP). In the ROC curve analysis, these three factors predicted the occurrence of FoP (AUC: 0.646, 0.703, and 0.658, respectively). Anxiety and depression played a parallel mediating role between sleep quality and FoP, accounting for 59.9 and 13.8% of the total effect, respectively.

**Conclusion:**

The results indicate that anxiety and depression both serve a parallel mediating role in the relationship between sleep quality and fear of pain (FoP). These findings provide potential guidance for the development and implementation of group-based interventions to address the mental health challenges of the post-COVID-19 era.

## Introduction

The COVID-19 pandemic caused by the SARS-CoV-2 virus is one of the defining public health emergencies of this century (Kokudo and Sugiyama, 2020). SARS-CoV-2 infection presents a range of clinical manifestations, from asymptomatic or uncomplicated upper respiratory tract infections to severe pneumonitis, multiorgan failure, and death (Herridge and Azoulay, 2022). China officially ended its zero-COVID-19 policy on 8th January 2023; this sudden shift caused the number of confirmed cases to increase rapidly (The Lancet Regional Health-Western Pacific, 2023). As of February 28, 2023, there have been a total of 758,390,564 confirmed cases worldwide, including 6,859,093 deaths (World Health Organization, 2023). The rapid outbreak of COVID-19 infection has instilled fear in populations (Reznik et al., 2021). Some studies indicate that more than 50% of individuals who have recovered from acute COVID-19 still experience symptomatic sequelae after 2 years, such as insomnia, dyspnea, muscle weakness, impaired fitness, mental fatigue, cognitive deficits, and affective symptoms (Bellan et al., 2021). Based on a systematic review that included 117 studies, about 12.2% of individuals who recover from COVID-19 become reinfected with the virus (Ren et al., 2021). Moreover, their quality of life remains reduced months after infection, as does their occupational performance (Lemhöfer et al., 2023). In addition, COVID-19 imposes a considerable economic burden on patients, families, other caregivers, and society (Álvarez-Del Río et al., 2023). Meanwhile, the prevalence of mental health problems, mainly fear of progression, anxiety, depression, insomnia, fatigue, and PTSD, has increased significantly in the general population during the COVID-19 pandemic, attracting widespread attention (Abel et al., 2021; Ding et al., 2022; Dorman-Ilan et al., 2020). The characteristics of the suddenness, severity, prevalence, and uncertainty of COVID-19 infection worldwide have further intensified fear in both infected and uninfected populations globally (Baby et al., 2022). Thus, it is crucial for healthcare professionals to examine the risk factors and underlying mechanisms related to fear of progression (FoP) in individuals recovering from COVID-19, as this information is significant for improving the physical and psychological discomfort of patients after recovery and enhancing their long-term quality of life.

Fear of progression refers to a logically understandable reaction to the genuine threat posed by a potentially life-threatening illness. It is characterized as the “fear that the illness will progress with all its biopsychosocial consequences or that it will recur” (Dankert et al., 2003). Proper fear is the body’s natural stress response when confronted with disease and may actively contribute to disease management. However, excessive and prolonged fear can reduce patients’ quality of life, social function, and self-reported happiness (Ban et al., 2021). Therefore, early detection and psychological intervention are necessary. Scholars have investigated the status quo and influencing factors of fear of progression (FoP) in patients with cancer, respiratory disease, Parkinson’s disease, diabetes, and chronic heart failure, finding that FoP is widespread and not optimistic in these patients (Folkerts et al., 2022; Liu et al., 2021; Wang Y. et al., 2022; Xiong et al., 2023). While FoP has been extensively studied in chronic illnesses, its relevance to COVID-19 survivors warrants distinct consideration. Unlike cancer patients, whose FoP is primarily driven by concerns about disease recurrence and treatment-related side effects, COVID-19 survivors grapple with distinct uncertainties, including the risk of reinfection, persistent symptoms—commonly known as long COVID—and the far-reaching psychological toll of the pandemic (Davis et al., 2023). Moreover, the unpredictability of viral mutations and the continued implementation of public health measures further exacerbate fear in this population (Markov et al., 2023). Given the large number of individuals affected by COVID-19 and the potential long-term health consequences, it is crucial to investigate FoP in this population to better inform psychological interventions and improve post-recovery wellbeing. To date, only one study has investigated FoP in patients with COVID-19, which found a high prevalence of FoP among participants (Ding et al., 2022). However, this study only analyzed the level of FoP in patients with COVID-19 infection and the related demographic factors, failing to conduct an in-depth analysis of the relationship between FoP and other relevant psychological variables. Therefore, exploring the factors influencing FoP in individuals recovering from COVID-19 is essential to offer guidance for reducing their FoP and promoting further adaptive growth. A multitude of domestic and international studies have investigated the effects of demographic and psychological factors on FoP in various patient groups. Through a literature review, it was found that the influencing factors of FoP were broadly categorized into demographic, disease-related, and psychosocial factors.

Previous studies revealed that there was a significant correlation between sleep quality and the development of FoP (Perndorfer et al., 2022). A study focusing on sleep disturbances in COVID-19 survivors found that the pooled prevalence of sleep disorders among these survivors was 32% (Wu et al., 2023). Poor sleep quality is associated with greater odds of having had a COVID-19 infection and significantly worsens the prognosis of the illness (Ahmet'yanov et al., 2022; Quan et al., 2023). Sleep disorders can induce delusions, metabolic disorders, hormonal disorders, immune interference, cardiovascular diseases, and respiratory diseases; these complications could compromise the overall prognosis of the survivors as well as the treatment effects (Bhat and Chokroverty, 2022). Importantly, insufficient sleep has been shown to blunt the immune response to vaccination (Rayatdoost et al., 2022) and increase the risk of developing rhinovirus infection (Prather et al., 2015). Research on neural mechanisms indicates that sleep deprivation facilitates fear acquisition by augmenting threat-specific encoding in the basolateral amygdala (Feng et al., 2023). During the COVID-19 pandemic, the FoP of patients with cancer was significantly associated with sleep dysfunction (Kim et al., 2022). Similar findings suggest that poor sleep quality is an independent risk factor for fear of falls among older people in the community (Zhang et al., 2023). While the majority of previous studies concentrated on cancer patients, the elderly, and community groups, individuals recovering from COVID-19 have not been surveyed. It is still uncertain whether poor sleep quality serves as a risk factor for FoP in individuals recovering from COVID-19. Therefore, to better understand the FoP of individuals recovering from COVID-19, the mediating mechanism through which sleep quality influences FoP requires investigation.

According to the stress responses theory (Compas et al., 2001; Meerlo et al., 2008), sleep difficulties may have an effect on an individual’s voluntary responses to stress (i.e., coping). Individuals experiencing sleep loss demonstrate a marked reduction in cognitive energy resources, which are necessary for selecting appropriate emotional regulation strategies in challenging circumstances (Zohar et al., 2005). In other words, chronically restricted and disrupted sleep may excessively activate stress systems, potentially leading to mood disorders such as anxiety and depression. Studies have established a significant positive relationship between sleep quality, anxiety, and depression (Palmer et al., 2018). Compared to healthy controls, individuals with symptoms of anxiety or depression report poorer sleep quality (Brenes et al., 2009). Additionally, anxiety is associated with increased sleep onset latency, which significantly impacts individuals’ subjective evaluations of sleep quality (Horváth et al., 2016). Evidence from a clinical study also supports the predictive effects of poor sleep quality on anxiety and depression (Zhu et al., 2023). Furthermore, relevant research indicates that anxiety and depression are significant predictors of fear of progression (FoP) in both Parkinson’s disease and cancer patients (Folkerts et al., 2022; Yang et al., 2018). Given these perspectives, it is reasonable to consider anxiety and depression as mediators between sleep quality and FoP.

The Transactional Model of Stress and Coping (Folkman et al., 1986) posits that when individuals encounter stressors, their adaptive outcomes are shaped by two key processes: cognitive appraisal and coping mechanisms. Cognitive appraisal consists of primary appraisal (evaluating whether a stressor poses a threat to wellbeing) and secondary appraisal (assessing available coping resources and strategies). According to this model, individuals interpret stressors and adopt coping strategies that ultimately influence psychological outcomes. In this context, poor sleep quality may act as a stressor, affecting fear of progression (FoP) through psychological mechanisms such as anxiety and depression. Drawing on these findings, we suggest that anxiety and depression may serve as mediating variables in the relationship between sleep quality and FoP in individuals recovering from COVID-19. Thus, we investigate the following hypotheses: Hypothesis 1: Poor sleep quality is related to higher FoP in individuals recovering from COVID-19. Hypothesis 2: Anxiety mediates the relationship between sleep quality and FoP in individuals recovering from COVID-19. Hypothesis 3: Depression mediates the relationship between sleep quality and FoP in individuals recovering from COVID-19. Concurrently, from the perspective of individuals recovering from COVID-19, we investigate the effect of sleep quality on FoP and assess the roles of anxiety and depression to offer potential guidance for developing and implementing group-based interventions to address mental health challenges in the post-COVID-19 era.

## Methods

### Participants

From January to February 2023, a convenience sampling method was employed to conduct a cross-sectional survey using anonymous self-administered questionnaires from outpatients at the Second Affiliated Hospital of Zhejiang University School of Medicine. Inclusion criteria were as follows: (a) participants aged 18 or older, (b) individuals recovering from COVID-19 who did not require supplemental oxygen or hospitalization, and (c) informed consent and voluntary participation in the study. The exclusion criteria included (a) the presence of cognitive impairment and/or diagnosed mental illness, (b) severe sensory impairments, including visual or hearing deficits, and (c) speech/language disorders that would impede communication. Participants were fully informed about the purpose and significance of the research and were invited to participate voluntarily.

### Procedures and measures

We used the N:q rule proposed by Jackson (2003) to calculate the sample size (Jackson, 2003). The N:q rule is appropriate for the maximum likelihood method, commonly used in structural equation modeling (Kline et al., 2011), where N represents the sample size and q refers to the number of items for the research variables. According to the guidelines, the optimal ratio of N to q should range from 10 to 20. With 68 parameters (20 for SAS, 20 for SDS, 16 for PSQI, and 12 for FoP), the ideal sample size should be between 680 (68 × 10) and 1,360 (68 × 20). To address sampling errors and invalid questionnaires, we distributed 900 questionnaires and received 866, yielding a response rate of 96.22%. After excluding five incomplete questionnaires, we obtained 861 valid responses, resulting in a valid response rate of 99.422%.

#### Pittsburgh sleep quality index (PSQI)

Sleep quality was assessed using the Pittsburgh Sleep Quality Index (PSQI) (Buysse et al., 1989). Sixteen of the 18 self-rated questions in this scale were scored individually, excluding the two questions related to bedtime and wake-up time. These questions covered seven dimensions: subjective sleep quality, sleep onset, sleep duration, sleep efficiency, sleep disturbance, hypnotic medication use, and daytime dysfunction. Higher scores indicated worse sleep quality. A total PSQI score above 7 was used to distinguish poor sleepers from good sleepers. The Chinese version of the PSQI demonstrated good internal consistency, with Cronbach’s *α* ranging from 0.82 to 0.83, and strong test–retest reliability, with a coefficient of 0.85 (Tsai et al., 2005). In this study, the Cronbach’s α coefficient was 0.801. The PSQI has been widely used among Chinese populations, and previous studies have provided preliminary evidence for its validity in Chinese clinical and general samples during the COVID-19 era (Hou et al., 2022; Wang L. et al., 2022; Zhang et al., 2021).

#### Self-rating anxiety scale (SAS)

We measured the anxiety of patients after COVID-19 infection using the Self-Rating Anxiety Scale (SAS), which was compiled by Zung (Dunstan and Scott, 2020). The scale consists of 20 items and employs a four-point Likert scoring system, ranging from 1 (never) to 4 (always). The cut-off point for anxiety is a standard score of 50, with higher scores indicating greater levels of anxiety. The Cronbach’s *α* coefficient for this study is 0.867. The SAS has been widely used in Chinese populations, and previous studies have provided preliminary evidence of its validity in Chinese clinical and general samples during the COVID-19 era (Du et al., 2024; Sun et al., 2022).

#### Self-rating depression scale (SDS)

The depression levels of patients after COVID-19 were measured using the Self-Rating Depression Scale (SDS), originally created by Zung (1965). This scale consists of 20 items and employs a four-point Likert scoring system, ranging from 1 (never or rarely) to 4 (most or all of the time). The cut-off point for depression is a standard score of 50, with higher scores indicating greater levels of depression. The Cronbach’s *α* coefficient for this study is 0.902. The SDS has been widely used in Chinese populations, and previous studies have provided preliminary evidence for its validity in Chinese clinical samples during the COVID-19 era (Chen L. et al., 2023).

#### Fear of progression questionnaire-short form (FoP-Q-SF)

The Fear of Progression Questionnaire-Short Form (FoP-Q-SF), developed by Mehnert et al. (2006) and translated by Wu et al. (2015), was used to measure the fear experienced by patients after COVID-19 infection. This scale consists of 12 items and two dimensions: physical health and social family. The items are scored on a five-point Likert scale, ranging from 1 (never) to 5 (regularly). A total score of 34 or higher indicates that the patient may have developed psychological dysfunction. The psychometric properties of the Chinese version of the FoP-Q-SF were tested by Wu et al. (2015), and the scale has been shown to be an effective tool with good reliability for measuring fear of progression in Chinese patients. The Cronbach’s *α* coefficient for this study is 0.913. The FoP-Q-SF has been widely used in Chinese populations, and previous studies have provided preliminary evidence for its validity in Chinese clinical samples during the COVID-19 era (Ding et al., 2022).

### Ethical considerations

This study received approval from the Ethics Committee of the Second Affiliated Hospital of Zhejiang University School of Medicine under approval number IR20230075.

### Statistical analysis

Data analysis was conducted using SPSS 21.0 and the PROCESS Macro. The Kolmogorov–Smirnov test was performed to assess the normality of continuous variables. Descriptive statistics were used to analyze the participants’ demographic characteristics and scale scores. A chi-squared test was used for binary data. The results of the univariate analysis, showing significant differences (*p* < 0.05), were entered into the regression model as independent variables for binary logistic regression analysis. Receiver operating characteristic (ROC) curves were used to assess the impact of sleep quality, anxiety, and depression on FoP. We assessed the linear relationships between continuous independent and dependent variables by examining scatter plots. Pearson’s correlation analysis was used to examine the relationships among four factors: sleep quality, anxiety, depression, and FoP. The PROCESS Model 4 (Hayes, 2013) was used to examine the mediating role of anxiety and depression between sleep quality and FoP among individuals recovering from COVID-19. Furthermore, to examine the effect of sleep quality on FoP, we calculated 95% confidence intervals for bias-corrected percentile bootstrapping through a bootstrapped sample of 5,000 (Taylor et al., 2008). The *p*-value is two-tailed, with a threshold of 0.05 for statistical significance.

## Results

### Sample characteristics and factors associated with FoP level

As shown in Table 1, a total of 861 individuals recovering from COVID-19 participated in the survey, including 272 men (31.6%) and 589 women (68.4%). In addition, 77.5% of participants were under 45 years old, while 22.5% were 45 years of age or older. The majority of participants were infected with SARS-CoV-2 within the last month (66.0%). Of the younger participants, 396 of them (59.4%) had low FoP as defined by Short FoP scores of less than 34 points, while the remaining participants were categorized as having high FoP.

**Table 1:** Comparison of sample characteristics between the low and high FOP groups (*N* = 861).

Variables		*N* (%)	Low FoP *n* (%)	High FoP *n* (%)	*χ^2^*	*p*
Age (years)	<45	667 (77.5)	396 (59.4)	271 (40.6)	5.978	0.014
	≥45	194 (22.5)	134 (69.1)	60 (30.9)		
Gender	Male	272 (31.6)	175 (64.3)	97 (35.7)	1.300	0.254
	Female	589 (68.4)	355 (60.3)	234 (39.7)		
Time after infection onset	≤1 M	568 (66.0)	366 (64.4)	202 (35.6)	5.851	0.016
	>1 M	293 (34.0)	164 (56.0)	129 (44.0)		
SQ	Good	191 (22.2)	161 (84.3)	30 (15.7)	53.620	<0.001
	Poor	670 (77.8)	369 (55.1)	301 (44.9)		
Anxiety	No	425 (49.3)	349 (82.1)	76 (17.9)	204.521	<0.001
	Mild	222 (25.8)	129 (58.1)	93 (41.9)		
	Moderate	150 (17.4)	41 (27.3)	109 (72.7)		
	Severe	64 (7.4)	11 (17.2)	53 (82.8)		
Depression	No	347 (40.3)	279 (80.4)	68 (19.6)	161.208	<0.001
	Mild	220 (25.5)	150 (68.2)	70 (31.8)		
	Moderate	166 (19.3)	72 (43.4)	94 (56.6)		
	Severe	128 (14.9)	29 (22.7)	99 (77.3)		

FoP, fear of progression; M, Month; SQ, sleep quality.

There were significant differences in FoP scores between the younger group and the older group (*p* < 0.05), with the proportion of high FoP among the younger group being significantly higher than that among the older group. Moreover, no significant gender differences were found between the two FoP groups. Regarding the time after infection onset, participants infected with SARS-CoV-2 for over 1 month in the high FoP group were significantly more numerous than those infected for less than 1 month (*p* < 0.05). In addition, the results indicated significant differences in sleep quality, anxiety, and depression between the high and low FoP groups (*p* < 0.05). Patients with poor sleep quality, severe anxiety, and severe depression had a higher incidence of FoP. Detailed information can be found in Table 1.

**Figure 1:**
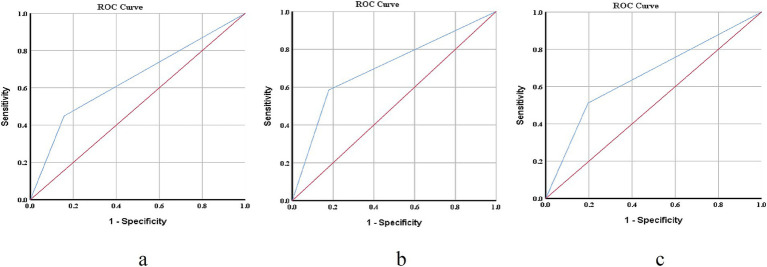
The ROC curves of each indicator in the prediction of FoP risk. **(a)** ROC curves of the prediction of PSQI total score to Fop, AUC = 0.646 ± 0.021, *p* < 0.001, 95%CI [0.605, 0.687]; **(b)** ROC curves of the prediction of SAS total score to Fop, AUC = 0.703 ± 0.018, *p* < 0.001, 95%CI [0.668, 0.738]; **(c)** ROC curves of the prediction of SDS total score to Fop, AUC = 0.658 ± 0.019, *p* < 0.001, 95%CI [0.621, 0.694]. ROC, receiver operating characteristic (ROC) curves; AUC, area under the curve (AUC); and FoP, fear of progression.

Based on the results of the univariate analysis, factors with *p* < 0.05 were selected for logistic regression analysis, including five variables: age, time after infection onset, sleep quality, anxiety, and depression. Binary logistic regression was then used to examine the independent effects of these factors on the fear of progression (FoP) in individuals recovering from COVID-19, with FoP as the dependent variable and the statistically significant factors as the independent variables. Retained in the model were sleep quality, anxiety, and depression (see Table 2). Figures 1a–c display the ROC curve and AUC for sleep quality, anxiety, and depression, respectively (AUC: 0.646, 0.703, 0.658, respectively). It should be noted that these three indicators are significant for predicting the incidence of FoP among the participants (*p* < 0.001).

**Table 2:** Analysis of potential factors associated with FoP in individuals recovering from COVID-19.

Variables	*β*	SE	Wald *χ^2^*	*p*	OR	95% CI
Age	−0.298	0.197	2.301	0.129	0.742	0.505, 1.091
Time from infection onset	0.140	0.171	0.677	0.441	1.151	0.824, 1.608
Sleep quality	0.513	0.238	4.654	0.031	1.670	1.048, 2.663
Anxiety	0.814	0.121	45.109	<0.001	2.258	1.780, 2.863
Depression	0.319	0.103	9.672	0.002	1.376	1.125, 1.682

### Descriptive analysis and correlations among overall variables

Table 3 presents the means, standard deviations (SD), and Pearson correlations for each variable. The participants’ PSQI scores were 9.75 ± 4.93, and 77.8% (*N* = 670) of the total participants had poor sleep quality, indicated by a global PSQI score of ≥5. The SAS, SDS, and FoP scores were 51.16 ± 12.06, 56.15 ± 14.84, and 30.72 ± 10.70, respectively. According to the cut-off point of each scale, 436 (50.6%), 514 (59.7%), and 331 (38.4%) participants were categorized as experiencing clinical anxiety, depression, and FoP, respectively. Pearson’s correlation analysis identified statistically significant positive associations between FoP and three key variables: PSQI scores (*r* = 0.380, *p* < 0.01), SAS scores (*r* = 0.578, *p* < 0.01), and SDS scores (*r* = 0.514, *p* < 0.01). Additionally, SDS scores were positively correlated with PSQI scores (*r* = 0.508, *p* < 0.01) and SAS scores (*r* = 0.814, *p* < 0.01). Furthermore, a significant positive correlation was also observed between SAS scores and PSQI scores (*r* = 0.552, *p* < 0.01). Detailed information can be found in Table 3.

**Table 3:** Means, standard deviations, and correlations of the study variables (*N* = 861).

Variables	1	2	3	4	5	6	7	8	9	10	11	12	13	Mean ± SD
1. PSQI	1													9.75 ± 4.93
2. SAS	0.552^**^	1												51.16 ± 12.06
3. SDS	0.508^**^	0.814^**^	1											56.15 ± 14.84
4. FoP-Q-SF	0.380^**^	0.578^**^	0.514^**^	1										30.72 ± 10.70
5. PSQI C_1_	0.786^**^	0.459^**^	0.454^**^	0.327^**^	1									1.66 ± 0.88
6. PSQI C_2_	0.728^**^	0.453^**^	0.444^**^	0.316^**^	0.632^**^	1								1.78 ± 1.05
7. PSQI C_3_	0.751^**^	0.324^**^	0.335^**^	0.195^**^	0.570^**^	0.417^**^	1							1.11 ± 1.06
8. PSQI C_4_	0.680^**^	0.190^**^	0.182^**^	0.084^*^	0.412^**^	0.347^**^	0.623^**^	1						0.73 ± 0.26
9. PSQI C_5_	0.652^**^	0.598^**^	0.483^**^	0.448^**^	0.493^**^	0.469^**^	0.378^**^	0.269^**^	1					1.41 ± 0.73
10. PSQI C_6_	0.591^**^	0.206^**^	0.139^**^	0.136^**^	0.300^**^	0.257^**^	0.305^**^	0.329^**^	0.260^**^	1				0.80 ± 1.23
11. PSQI C_7_	0.618^**^	0.545^**^	0.512^**^	0.426^**^	0.472^**^	0.438^**^	0.283^**^	0.150^**^	0.482^**^	0.226^**^	1			1.91 ± 1.05
12. Fop-PH	0.407^**^	0.551^**^	0.472^**^	0.947^**^	0.352^**^	0.325^**^	0.225^**^	0.119^**^	0.464^**^	0.158^**^	0.417^**^	1		16.27 ± 5.75
13. Fop-SF	0.310^**^	0.542^**^	0.500^**^	0.943^**^	0.266^**^	0.271^**^	0.142^**^	0.039	0.382^**^	0.098^**^	0.387^**^	0.786^**^	1	14.45 ± 5.57

PSQI, Pittsburgh Sleep Quality Index; SAS, Self-Rating Anxiety Scale; SDS, Self-Rating Depression Scale; FoP-Q-SF, Fear of progression questionnaire-short form; PSQI C_1_, Subjective sleep quality; PSQI C_2_, Sleep latency; PSQI C_3_, Sleep duration; PSQI C_4_, Habitual sleep efficiency; PSQI C_5_, Sleep disturbance; PSQI C_6_, Use of sleep medication; PSQI C_7_, Daytime dysfunctions; FoP-PH, Fear of progression-Physiological health; and FoP-SF, Fear of progression-Social family.

^*^p < 0.05; ^**^p < 0.01.

### Mediating effect analysis

Logistic regression analysis revealed that age and time after infection onset had significant effects on the FoP of individuals recovering from COVID-19. Therefore, these variables were used as control variables in the mediating effect analysis.

**Figure 2:**
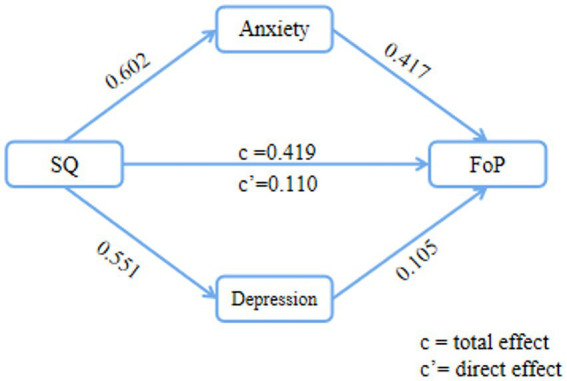
The mediating effect of anxiety and depression between sleep quality and FoP.

Then, the SPSS PROCESS Macro Model 4 was employed to analyze the mediation effect, with the results presented in Table 4 and Figure 2. The results showed that, first, sleep quality significantly and positively predicted FoP (*β* = 0.419, *p* < 0.001, Model 1). Second, sleep quality also significantly and positively predicted anxiety (*β* = 0.602, *p* < 0.001, Model 2). Third, sleep quality significantly and positively predicted depression (*β* = 0.551, *p* < 0.001, Model 3). Fourth, when sleep quality, anxiety, depression, and FoP were entered into the regression equation concurrently (Model 4), the predictive effect of sleep quality remained significant (*β* = 0.110, *p* < 0.001). Both anxiety and depression significantly predicted FoP (*β* = 0.417, *p* < 0.001; *β* = 0.105, *p* < 0.001), indicating that anxiety and depression play a mediating role between sleep quality and FoP.

**Table 4:** Regression analysis of the relationship between variables in the mediation effect model (*N* = 861).

Predictive variable	Model 1 (criterion: FoP)		Model 2 (criterion: Anxiety)		Model 3 (criterion: Depression)		Model 4 (criterion: FoP)	
	*β*	*t*	*β*	*t*	*β*	*t*	*β*	*t*
Age	−0.304	−6.824^***^	−0.322	−8.086^***^	−0.286	−6.886^***^	−0.139	−3.369^***^
Time from infection onset	0.128	2.229^*^	0.020	0.388	0.037	0.694	0.116	2.254^*^
Sleep quality	0.419	13.179^***^	0.602	21.143^***^	0.551	18.581^***^	0.110	3.113^*^
Anxiety							0.417	8.402^***^
Depression							0.105	2.207^*^
*R* ^2^	0.193		0.354		0.298		0.356	
*F*	68.412		156.404		121.092		94.661	

^*^p < 0.05; ^***^p < 0.001.

Finally, the bootstrap method with percentile bias correction was employed to examine the mediating effect of anxiety and depression between sleep quality and FoP. The results indicated that the mediating effect of anxiety and depression was significant, with a total indirect effect value of 0.309, representing 73.75% of the total effect (0.419). In other words, anxiety and depression mediate 73.75% of the relationship between sleep quality and FoP. Specifically, the mediating effect includes indirect effects through two pathways. The first pathway demonstrated that the indirect effect of sleep quality on FoP through anxiety had a path coefficient of 0.251 (Bootstrap 95% CI: 0.005, 0.112). Second, the path coefficient of the indirect effect of sleep quality on FoP through depression was 0.058 (Bootstrap 95% CI: 0.005, 0.111). The total effect, direct effect, and indirect effect sizes are shown in Figure 2.

## Discussion

This study explored the causal association among sleep quality, anxiety, depression, and FoP. Among the participants, the sleep quality score was higher than the results reported by Kalamara et al. (2022) and Hou et al. (2022). The possible reasons are as follows: Our study was conducted from January to February 2023, shortly after the end of the zero-COVID-19 policy, when infections were widespread, significantly impacting the mental health of the general population. Alternatively, it may be due to age, cultural, and other differences. By evaluating overall sleep quality with the aid of the Pittsburgh Sleep Quality Index (PSQI), our study revealed that 77.8% of participants reported sleep disturbances, which is similar to previous research indicating that 50–75% of COVID-19 patients experience sleep deterioration (Alimoradi et al., 2021; Jahrami et al., 2021; Pataka et al., 2021). While this proportion is slightly higher than that of breast cancer patients (Cho and Hwang, 2021; Weng et al., 2021). The participants’ anxiety score was mild, which was lower than the findings of Sun et al. (2021); this difference may be due to their research participants being COVID-19 patients in quarantine wards. Furthermore, the participants’ depression score was generally mild. The prevalence of depression, assessed using the SDS scale, revealed that approximately 59.7% of respondents exhibited symptoms of depression. Our result regarding the prevalence of depression was higher than the research by Khatun and Farhana (2023). Furthermore, the results indicated that FoP in individuals recovering from COVID-19 was at a moderate level, with 38.4% of participants showing clinically significant FoP, suggesting psychological dysfunction. This finding aligns with research by Ding et al. (2022). However, this proportion is lower than that of cancer patients (Chen R. et al., 2023; Liu et al., 2024). This difference may stem from variations in disease characteristics; the sudden infectious nature of COVID-19 and the long-term threat of cancer have distinct psychological impacts on patients. FoP can affect both short-term and long-term clinical outcomes, cause distress when reintegrating into society and family, and affect patients’ quality of life, highlighting the need for increased attention from healthcare professionals.

The score of the FoP physical health dimension for COVID-19 patients in our research is slightly higher than that of the FoP social family dimension, suggesting that medical staff should implement targeted psychological nursing interventions and disease-related health education in advance to reduce participants’ anxiety about disease progression. The study revealed that the proportion of individuals with high FoP was significantly higher in the under 45-year-old group compared to the group aged 45 years and older, consistent with previous studies on cancer patients (Hinz et al., 2015; Hu et al., 2024). Younger individuals may face greater uncertainty regarding their future health, career trajectories, and social reintegration during the pandemic, which could contribute to heightened fear of progression (FoP). Additionally, their increased exposure to health-related information through social media may amplify concerns about long-term health consequences, the risk of reinfection, and potential post-viral complications. Furthermore, social and economic stressors, such as job instability and financial burdens during the recovery period, may further exacerbate FoP in this demographic. Our study found that low sleep quality, anxiety, and depression were independent risk factors for high FoP. Poor sleep quality is related to higher FoP in individuals recovering from COVID-19, which supports our Hypothesis 1 and is consistent with Yang’s view (Yang et al., 2022). Individuals with sleep difficulties tend to experience excessive negative intrusive thoughts during the day, including worries about the future. In particular, poor sleep may aggravate participants’ anxiety, uncertainty, and worries about their prognosis, long-term effects of treatment, or relapse (Rensen et al., 2019). Thus, individuals recovering from COVID-19 with poor sleep quality are at a higher risk of experiencing elevated levels of FoP. This finding suggests that improving sleep quality could serve as a potential approach to controlling or reducing FoP in these individuals.

Furthermore, the receiver operating characteristic (ROC) analysis demonstrated that anxiety, depression, and sleep quality demonstrate predictive ability for FoP, with AUC values of 0.646, 0.703, and 0.658, respectively. Depression (AUC = 0.703) shows acceptable predictive ability for FoP, indicating that individuals with higher depressive symptoms are significantly more likely to experience heightened fear of progression. Meanwhile, sleep quality (AUC = 0.658) and anxiety (AUC = 0.646) exhibit poor-to-fair predictive power, suggesting that while these factors contribute to FoP, their predictive ability alone is relatively limited. Given the poor-to-fair predictive ability of sleep quality and anxiety, these factors should not be overlooked but rather considered in combination with other psychological indicators when assessing individuals at risk for elevated FoP. From a clinical nursing perspective, these results highlight the need for early psychological screening and intervention in post-COVID recovery programs. Since depression shows the strongest predictive power, targeted mental health support—such as psychotherapy, cognitive-behavioral interventions, and emotional support programs—may help mitigate FoP. Additionally, interventions aimed at improving sleep hygiene and anxiety management could serve as complementary strategies to enhance psychological resilience. Overall, while the AUC values indicate that anxiety, depression, and sleep quality play significant roles in FoP, their moderate effect sizes suggest that a multifactorial approach is necessary for comprehensive assessment and intervention. Future studies should explore additional factors, such as coping strategies and social support, to improve predictive accuracy and develop more effective intervention frameworks.

In this study, Pearson’s correlation analysis revealed weak to strong positive associations among fear of progression (FoP), sleep quality, anxiety, and depression, with correlation coefficients ranging from *r* = 0.380 to *r* = 0.814. The correlation between FoP and PSQI scores (*r* = 0.380) suggests a weak association, indicating that poorer sleep quality is weakly related to higher FoP. The correlations of FoP with SAS (*r* = 0.578) and SDS (*r* = 0.514) indicate a moderate association, suggesting that anxiety and depression are moderately associated with FoP. The strong correlation between SAS and SDS scores (*r* = 0.814) indicates a high degree of association between anxiety and depression, a well-documented phenomenon in mental health research. This reinforces the notion that individuals experiencing heightened anxiety are likely to also experience depressive symptoms, further complicating their psychological recovery process. The moderate correlations between SDS and PSQI scores (*r* = 0.508) and SAS and PSQI scores (*r* = 0.552) suggest that sleep disturbances are closely linked with both anxiety and depression. The strong correlation between anxiety and depression suggests that interventions should not target these conditions in isolation but rather take a holistic approach to mental health management. Furthermore, given the weak association between sleep quality and FoP, improving sleep hygiene and addressing insomnia-related issues may serve as an indirect yet meaningful strategy for reducing FoP. From a nursing and healthcare perspective, these results highlight the necessity of integrated psychological support in post-COVID rehabilitation programs. Screening for anxiety, depression, and sleep disturbances should be incorporated into routine follow-ups, and interdisciplinary interventions involving mental health professionals, sleep specialists, and nursing care providers should be considered to enhance overall wellbeing and recovery outcomes.

This study found that anxiety and depression partially mediated the association between sleep quality and the fear of progression (FoP). These results support Hypothesis 2 and Hypothesis 3. Previous research has shown that sleep disturbance is a strong predictor of subsequent affective disorders, including anxiety, depression, and suicide (Ding et al., 2023; Um et al., 2023). Studies conducted among cancer patients have yielded similar results (Cho and Hwang, 2021). In other words, participants with poor sleep quality were more likely to experience increased anxiety and depression (Yiyue et al., 2023), which further aggravates the level of fear of disease progression, thus forming a vicious circle (Wang X. et al., 2022). On the one hand, studies have pointed out that anxiety and depression can significantly inhibit the cellular immunity of individuals (Zhou et al., 2005). As stressors, anxiety and depression can affect neurotransmitter and hormone levels, causing a decline in collective immune function and leading to increased fear about physical health among patients (Zhang et al., 2022). Furthermore, due to the characteristics of rapid transmission, multiple transmission channels, strong infectivity, and general susceptibility of the public, COVID-19 can generally cause individuals to worry about the burden it brings to their families and even to society, while anxiety and depression exacerbate this concern (Nakhaee Moghadam et al., 2022). Higher anxiety or depression may worsen prognosis by negatively impacting participants’ immunity, potentially leading to significant consequences for their quality of life. Moreover, anxiety and depression can lead to reduced social participation (Kampf et al., 2016), increased cognitive impairment, and impaired quality of life (Baldiotti et al., 2023). Previous research in cancer patients suggests that higher anxiety and depression levels are key predictors of FoP, emphasizing the role of emotional distress in amplifying fears about disease progression (Jrad et al., 2024).

A novel finding of this study is that anxiety and depression partially explain the connection between sleep quality and FoP. While causal conclusions cannot be drawn from this study, the influence of anxiety and depression on the relationship between sleep quality and FoP is significant. Our findings suggest that interventions targeting sleep quality may be associated with lower FoP levels, potentially through pathways linked to anxiety and depression. However, experimental or longitudinal studies are required to confirm these relationships.

## Limitations

The study has several limitations. First, as a cross-sectional study, it only examined the relationship between variables at a single point in time and could not establish their dynamic development or causal relationships. Future research should consider employing longitudinal designs to understand the dynamic changes in sleep quality, anxiety, depression, and fear of progression (FoP) over time and to establish causal relationships between these variables. Due to data constraints, we could not control for all confounding variables. Investigating potential moderating factors, such as social support and resilience, could provide deeper insights into their influence on the observed relationships. Secondly, the participants in this study were from a single hospital, which limits the generalizability of the findings. Future studies could conduct broader research across various types of hospitals in different regions to enhance the generalizability of the results. Finally, since the data were self-reported, recall bias could impact the results. Although no method bias was detected in this study, future research should employ diverse data collection methods, such as combining self-reports with reports from others, to improve the reliability of the conclusions.

## Conclusion

This study investigated the sleep quality, anxiety, depression, and fear of pain (FoP) of individuals recovering from COVID-19. From the perspective of individuals recovering from COVID-19, the study developed a mediation model to examine how sleep quality influences participants’ FoP. The study discovered that anxiety and depression played parallel mediating roles in the effect of sleep quality on participants’ FoP. These findings provide potential guidance for the development and implementation of group-based interventions to address the mental health challenges of the post-COVID-19 era. Specifically, cognitive-behavioral therapy (CBT)-based interventions might be effective in addressing the psychological distress identified in our study. Additionally, we recommend sleep hygiene programs that focus on establishing regular sleep–wake cycles, reducing screen exposure before bedtime, and improving sleep efficiency. Given the strong interconnections among sleep, anxiety, depression, and FoP in COVID-19 survivors, integrating these approaches into post-recovery care may help mitigate psychological distress and improve overall wellbeing.

## Data Availability

The raw data supporting the conclusions of this article will be made available by the authors without undue reservation.
